# About the Biodiversity of the Air Microbiome

**DOI:** 10.32607/actanaturae.11671

**Published:** 2022

**Authors:** N. B. Naumova, M. R. Kabilov

**Affiliations:** Institute of Chemical Biology and Fundamental Medicine, Siberian Branch of the Russian Academy of Sciences, Novosibirsk, 630090 Russia

**Keywords:** bioaerosol, microbiome, troposphere, atmospheric transport, biodiversity

## Abstract

This brief review focuses on the properties of bioaerosols, presenting some recent results of metagenomic studies of the air microbiome performed using next-generation sequencing. The taxonomic composition and structure of the bioaerosol microbiome may display diurnal and seasonal dynamics and be dependent on meteorological events such as dust storms, showers, fogs, etc., as well as air pollution. The Proteobacteria and Ascomycota members are common dominants in bioaerosols in different troposphere layers. The microbiological composition of the lower troposphere air affects the composition and diversity of the indoor bioaerosol microbiome, and information about the latter is very important, especially during exacerbated epidemiological situations. Few studies focusing on the bioaerosol microbiome of the air above Russia urge intensification of such research.

## INTRODUCTION

Microorganisms are found ubiquitously in the environment and play a crucial role in almost all ecosystems [1]. Since many pathogens spread through the airborne route, including the SARS-CoV-2 coronavirus that has caused the current COVID-19 pandemic, it is especially relevant to study, monitor, and control the composition of outdoor and indoor air [2, 3]. Much data have been gained about the correlation between outdoor air pollution and the more severe course of COVID-19: for example, in India, a lower mortality rate from COVID-19 was observed in cities with better air quality [4]. We would like to emphasize that the term “bioaerosol” covers a broad range of particulate organic matter contained in the atmosphere, originating from various living and dead organisms [5]. Along with particulate matter of microbial, plant, or animal origin, bioaerosols usually also contain a broad range of antigenic compounds, microbial toxins, and viruses [6, 7]. Understanding the processes of bioaerosol formation, their distribution patterns, migration, structure, etc., especially under the harsh conditions of the upper atmosphere, is required for many fundamental and applied scientific disciplines [8], such as physics, chemistry, meteorology, and atmospheric hydrology; research into the content of allergy-inducing particles and microorganisms pathogenic to humans, farm animals, and plants; as well as aerobiology, biogeography, biodiversity, and general ecology. The key trends in bioaerosol research include (a) assessment of their sources and flows, (b) spatial distribution and its changes over time, (c) aging of biological particles, (d) metabolic activity, (e) urbanization of allergies, (f) pathogen transport, and (g) the impact on climate [8].

This review aims to briefly describe the bioaerosol microbiota, with special focus placed on the microbiome composition and structure. Air is an extremely dynamic (and, therefore, very challenging) environment for collecting and analyzing bioaerosol samples, identifying the aerosolization sources and transport pathways, so the methodological aspects of sample collection are undoubtedly of great significance for data interpretation and comparison. The microbiome analysis techniques are also very important. Nevertheless, since these two trends are rather extensive, we will touch upon them only briefly in this review.

## THE MAIN PROPERTIES OF BIOAEROSOLS

Bioaerosols are an important component of atmospheric aerosols. Calculations show that bioaerosols account for 10–28 vol.% [9] and 16–80 wt.% of all the particulate matter found in the air [1].

**Fig. 1:**
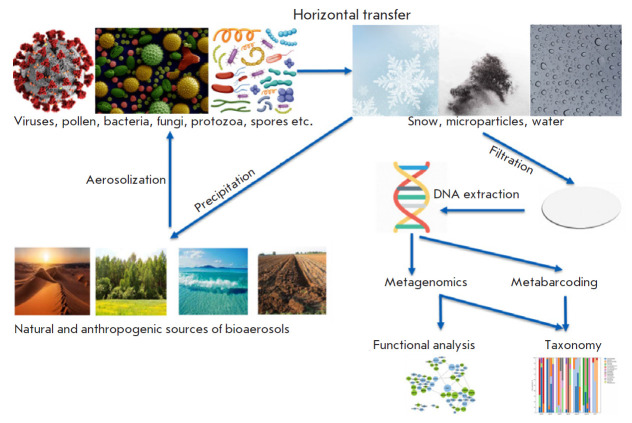
Bioaerosol formation, distribution, and analysis: schematic presentation

The airborne transmission of microorganisms is ubiquitous, being an essential part of the life cycle for some of them [10]. Various natural sources such as soil, forests, deserts, oceans, seas, etc. [11], as well as anthropogenic ones (agriculture, food industry, landfills, etc.), contribute to bioaerosol formation (Fig. 1) [6, 11, 12].

Once microorganisms get into the atmosphere (i.e., are aerosolized), they are much more likely to be exposed to the stress caused by drying, UV radiation, low temperatures, low carbon content, and low energy compared to their natural habitats (the sources of aerosol): so, many microorganisms may die [13].

The size of bioaerosol particles varies from 3 nm [14] to 100 μm, depending on their source: the diameter of pollen is 17–58 μm; that of fungal spores, 1–30 μm; the diameter of bacterial cells usually is 0.25–8 μm [15]; and that of viruses, < 0.3 μm. Meanwhile, the biological material does not necessarily consist of individual particles: most bacteria are associated with particles with a diameter > 2 μm [16, 17], 2–3 μm [18], and 3–4 μm [19, 20]. In some cases, bacteria were found to be characterized by a bimodal bioaerosol particle size distribution with the peaks at 1–2 μm and 4–7 μm [21]. Bacteria can also occur as cell agglomerates or be associated with plant, animal, or soil particles, as well as pollen or spores. Airborne bacterial cells and fungal spores can have concentrations as high as ~ 103÷104 and ~105 per m3 [17, 21] and be found at altitudes up to 40 km above sea level; i.e., up in the stratosphere [22]. In the near-surface layer of the troposphere, the concentration of bacterial particles capable of forming colonies on laboratory culture media ranged from 65 to 355 CFU/m3 in urban areas in southern Poland [19] and from 300 to 1350 CFU/m3 in urban and rural areas in Thailand [18]. In the latter case, the number of CFUs decreased rapidly with altitude (twofold when proceeding from 1–3 m to 7 m above ground level). Laboratory cultivation revealed that spore-forming bacteria Bacilli/ Firmicutes were significantly dominant in the near-surface and higher layers (several thousand meters) of the troposphere over the south of West Siberia [23, 24], while non-spore-forming bacteria were dominant over the northern part of this region [25].

Bioaerosol distribution in the air depends on the particular season [19, 26]. Thus, the concentration of bacterial cells in the air of the coastal region of China determined microscopically was higher in winter than in summer [21]. The bioaerosol load with pathogenic microbiota can vary greatly depending on the time of year: in South Asia, the pathogen content was found to substantially rise during the post-monsoon season and winter. Significant diurnal variation in bioaerosol composition was also detected [26].

Temperature and ultraviolet radiation are the most statistically significant meteorological factors responsible for the viability of airborne bacteria [19, 21]. The aerosol load with biota and their behavior in the environment largely depend on air pollution (haze, fog, dust, and various macroparticles), including pollution from transportation and biomass burning [26]. The proportion of viable bacteria in the total pool depends on the degree of pollution [15]. The bioaerosol composition can vary depending on specific, random meteorological conditions: for example, dust storms strongly increased the concentration of microorganisms in bioaerosols [16], and different bioaerosol components vary in different ways depending on meteorological conditions.

The data obtained so far indicate that bioaerosols play an important role [6, 11, 27, 28] in the physical and chemical processes occurring in the atmosphere [1, 29]. It was shown that bioaerosols can bind to surrounding particles, thus influencing atmospheric processes by acting as condensation nuclei in clouds and initiating precipitation [10, 30, 31]. Thus, it was found that biological particles act as nuclei for snow and cloud formation in 33% of cases [32].

Along with having an impact on weather phenomena, bioaerosols also affect human health [33], since they may contain pathogenic or opportunistic bacteria, fungi, viruses, high-molecular-weight allergens, bacterial endotoxins, mycotoxins, peptidoglycans, β(1-3)- glycans, pollen, and plant fibers [6]. First, the unfavorable effects of bioaerosols on human health manifest themselves as respiratory symptoms. Thus, there is a strong correlation between the increased outdoor air pollen concentration in spring and summer and asthma exacerbation in children [34]. An association was found between the content of fungal spores in the air and the number of patients with asthma symptoms requesting medical assistance [35]. The endotoxin of bacterial bioaerosols is considered an important etiological factor of occupational lung diseases, including non-allergic asthma [6]. Escherichia coli isolates, which are commonly used as water quality indicators, have also been found in atmospheric dust [36].

## AEROSOL SAMPLE COLLECTION

Aerosol sample collection is based on various physical approaches to separating particles from the air flow [37]. But the general idea is to pump air through a filter or a fluid medium, entrapping aerosol particles [38]. Techniques allowing one to separate particles according to their size during sample collection have recently started to appear [39]. They are particularly relevant for aerovirology: over the past decade, there has been intense research into the methods that can be used to collect indoor aerosol samples to monitor the effects of human breathing. In general, the instrumental options of aerosol collection have not been standardized yet and vary widely but the general principle of how they operate remains unchanged.

## METAGENOMIC SEQUENCING

The current research into the taxonomic diversity of the microbiota in bioaerosols relies on the approaches employing next-generation sequencing. Total DNA is extracted from the total pool of microorganisms trapped in a filter or a liquid medium and further used in a metagenomic analysis. As such methods are being developed, it has become possible to identify the unculturable microorganisms that are the major component of the aerial biome [40]. The metagenomic findings obtained thus far have shown that the dominant species of microorganisms identified using this technique differ from those identified by conventional culturing methods [41], since > 99% of the microorganisms detected in the air cannot be grown under laboratory conditions [26]. The term “microbiome” has been coined and has become widely used; the following definition was provided in the Microbiome journal: “This term refers to the entire habitat, including the microorganisms (bacteria, archaea, lower and higher eukaryotes, and viruses), their genomes (i.e., genes), and the surrounding environmental conditions” [42]. However, in publications using the term “microbiome” in their titles or keywords, there is often a mismatch with this definition, since most studies focus on a single group (viruses, bacteria, fungi, or plants), or, in the best-case scenario, on a combination of two groups. Without going deep into the reasons for this state of affairs, in this review we only mention this fact and emphasize that when further using the term “microbiome,” we mean the bacterial or fungal components of the microbiome or their combination, in line with the authors of the cited studies.

Hence, one can find a large number of publications related to research into the microbiomes in all types of natural objects, such as hot springs, lakes, seas, soil, the endogenous microbiota of organisms, etc. [43, 44, 45, 46]; however, catastrophically few publications have focused on the metagenomic analysis of bioaerosols [47, 48, 49, 50, 51, 52].

Metagenomic sequencing can be conveniently divided into two global directions: whole-genome metagenomic analysis and targeted sequencing (metabarcoding). In the former case, the entire DNA isolated from the sample is read, which allows one to talk about the taxonomic diversity, while on the other hand offering an opportunity to analyze its functional properties. However, the cost of the metagenomic approach is surely higher [48, 53] than that of metabarcoding, which is based on analyzing the highly conserved marker genes such as 16S (bacteria and archaea), ITS (fungi and plants), rbcL (plants), 18S (various eukaryotes), etc. [54, 55]. Meanwhile, efficiency in taxonomic identification depends directly on the number of verified sequences in the specialized databases being used. Today, the most comprehensive databases are those for prokaryotes (16S) and fungi (ITS).

The exceptionally low content of microorganisms in the air, along with the significant variation in the composition of microbial ensembles, poses a serious problem in analyzing the biodiversity, the function spectrum, and metabolic activity of bioaerosol microbiota [56]. We would like to emphasize that studies of this type are a fundamental basis for identifying aspects of human–nature interactions, and in particular those related to the routes of disease transmission and potential impact on human health [57]. Nevertheless, only sporadic results of the metagenomic analysis of bioaerosols have been reported in Russia [58].

**Bacterial microbiome in bioaerosols**

In the near-surface layers of the atmosphere, bacteria constitute a significant portion of bioaerosols: for example, in the Colorado mountains (USA), the average bacteria content among aerosol particles sized > 0.5 μm was 22% [47].

Aerosol bacteria can have a significant impact on the atmospheric chemistry, thus affecting human health [15]. For example, high air pollution levels can greatly alter the structure of the bacterial microbiome in humans [59]. On foggy days in Beijing, the contents of pathogenic Halomonas and Shewanella bacteria were found to increase [60], especially in autumn and early winter.

Metagenomic sequencing has revealed that the bacterial microbiome in bioaerosols is substantially biodiverse [61]. For example, 38 bacterial taxa were identified in the near-surface layers of the troposphere in urban areas [41]. Most studies demonstrated that Proteobacteria, Firmicutes, and Actinobacteria are the major phyla in the bacterial microbiome of the lower [41, 62, 63] and upper troposphere [50, 64, 65]. Meanwhile, in the lower troposphere in urban areas, Firmicutes can make a significant contribution (20–30%), while such phyla as Cyanobacteria, Bacteroidetes, Chloroflexi, Acidobacteria, and Deinococcus-Thermus are the minor phyla (1–5% of the relative content of nucleotide sequences). However, other studies showed a high proportion of the Bacteroidetes phylum members in bioaerosols in the air above Japan after dust storms in Asia [17, 66], as well as in the air above eastern Australia [67]. The composition of the bacterial microbiome in the upper troposphere above the Noto Peninsula in Japan was quite specific, where it was demonstrated (although using fluorescence in situ hybridization) that 80% of all eubacteria on mineral aerosol particles were represented by Bacillus subtilis belonging to the phylum Firmicutes [68].

Various weather events have a significant impact on the composition and structure of the bacterial microbiome of bioaerosols. For example, the long-distance transport of dust particles aerosolized during dust storms by air currents over seas and continents is an important mechanism for the introduction of various microorganisms into local ecosystems [69]. Thus, storms in the Sahara Desert cause the penetration of dust particles into the atmosphere, which are then transported to Europe together with air masses; in particular, this leads to their accumulation in the Alpine snow at an altitude of > 3,000 m above sea level [70]. Bioindicators of dust particles transferred from Algeria were members of the phyla Gemmatimonadetes and Deinococcus-Thermus [70], which are known to occur in dry oligotrophic habitats with relatively high levels of solar radiation; it allows them to survive during the transfer, while maintaining their metabolic activity. Very small quantities of pathogenic bacteria can be transferred with dust particles over very long distances [70]. The human body surface is a more plausible (compared to other biotopes) source of pathogenic bacteria in the air [71]. It was revealed that there is a clear dependence between the structure and composition of the bacterial microbiome at an altitude of 10 m above ground level (an island and a peninsula in East Asia) and dust storms in Central Asia [69]. Meanwhile, dust particles act as ice nucleation centers [72]. Precipitation is another important mechanism of transferring microorganisms from the upper to the lower troposphere, as well as to the terrestrial surface [73]. This study has shown that the composition of the bacterial microbiome in precipitation (a) corresponded to the bioaerosol sources along the transfer route and (b) exhibited an obvious seasonal dynamics when the relative abundance of prevailing Proteobacteria decreased from summer to winter.

It is noteworthy that, as opposed to the mycobiome, whose indoor composition depended on its outdoor composition and was independent of people’s activity indoors, the indoor biodiversity of the bacterial microbiome was dependent both on the outdoor bacterial microbiome [65] and on people’s activity indoors [41]. However, outdoor air pollution may not affect the biodiversity of bacterial and archaeal ensembles in indoor bioaerosols, as it was shown in a study conducted in Beijing [74]. This indicates that there are different mechanisms of formation and dynamics of different microbiome components, which should be borne in mind when planning observational experiments.

Cyanobacteria, which cause various health problems when they are inhaled, may contribute substantially to the total load with airborne particles [75]. Picocyanobacteria were recently detected in the near-surface atmospheric layers above land or water bodies in Greenland and Antarctica [76], where soil and water aerosolization is the leading mechanism of aerosol formation. Their transfer by wind is considered to be the main source of Cyanobacteria in air.

A meta-analysis of the results of 42 studies, covering more than 3,000 bioaerosol samples, revealed increased bacterial diversity, and relative abundance of pathogens in the samples associated with anthropogenic activity at collection sites [71].

**Mycobiome of bioaerosols**

Aerosol mycobiomes vary greatly; however, at the phylum level, Basidiomycota and Ascomycota are the major components of the mycobiome in both the near-surface and higher troposphere layers (they can switch places in terms of dominance). Thus, the members of the phylum Ascomycota were dominant (more than two-thirds) in the near-surface air layer in the Colorado mountains at an altitude of > 3,000 m above sea level [77], as well as in the near-surface air layers in Kuwait at a significantly lower altitude [78]. Other researchers, however, revealed that the phylum Basidiomycota was dominant (≥ 60%) [41, 63, 79], while the phylum Ascomycota accounted for about one-third of the fungal sequences. Interestingly, the proportion of members of the phylum Ascomycota (Cladosporium and Alternaria) resistant to atmospheric stress increased with altitude (500–800 m vs. 5–10 m) over the Gobi and the Taklimakan Deserts [80], which are the key suppliers of dust particles to the Asian atmosphere. In the near-surface air above a 3,043-m high mountain in Austria, members of the phylum Basidiomycota (Agaricomycetes) were dominant, followed by members of the phylum Ascomycota such as Dothideomycetes, Saccharomycetes, Sordariomycetes, Leotiomycetes, and Eurotiomycetes [64]. Ascomycetes, members of the family Davidiellaceae, accounted for 25% of the mycobiome in the direction from northeastern China towards Japan [81]. However, a recent study addressing fungal biodiversity in aerosols over Antarctica detected no members of this family among the dominant families of the mycobiome [82]. The fungus Alternaria, belonging to Pleosporaceae/ Pleosporales/Dothideomycetes/Ascomycota, is often identified among the major dominant fungal species of surface air layers both in urban areas (Nanjing, Beijing, and Seoul) and under natural conditions (the desert in Kuwait) [41, 78, 83]. The cultivated fungal genera Alternaria, Aspergillus, Penicillium, Cladosporium, etc., which are well-known as the major components of aerosol mycobiota [84], may account for ≤ 12% of the total number of marker nucleotide sequences in the metagenomic approach [41]. It should be borne in mind, however, that the relative content of Alternaria in the air can vary greatly (from 10 to 40%) over both rural and urban areas depending on the year [85]. A relationship between the mycobiome composition of near-surface aerosols and the vegetation type and condition (humidity of leaves) was revealed in the same study. Some papers describe a quite unexpected mycobiome composition (i.e., the one significantly differing from the data reported in other studies). Thus, the sequences of the genus Candida (Saccharomycetales/Saccharomycetes/Ascomycota) were shown to account for 54% of the mycobiome of the lower troposphere [81]. As for the near-surface layer, it was revealed in the same study [81] that the mycobiome consisted exclusively of Aspergillus spp. (Aspergillus/Aspergillaceae/Eurotiales/Eurotiomycetes/ Ascomycota). It is obvious that the composition of indoor bioaerosols largely depends on that of the near-surface atmospheric outdoor air, being especially true for the mycobiome whose composition depended on that of outdoor bioaerosols and was virtually independent of human activity, as has been demonstrated in a study of indoor air in kindergartens conducted in Korea [41]. The diversity of the indoor mycobiome may depend on outdoor air pollution, as was shown in a study conducted in Beijing [74]. Similar to the bacterial microbiome, the mycobiome composition may vary depending on particular meteorological events: for example, the content of fungi belonging to the class Agaricomycetes/Basidiomycota [86], which release vast quantities of spores into the atmosphere after rains, increased significantly after a rain over the arid area of the Mediterranean.

## CONCLUSIONS

Hence, the bioaerosol microbiome is a highly dynamic system. Variation in the microbiome composition and structure depends on a vast array of factors. Many of them mediate, disguise, or interfere with each other, thus preventing one from identifying unambiguous spatial and temporal regularities. Transfer of microorganisms over long distances by air currents in the upper troposphere has a crucial impact on the composition of the lower layers that humans are directly in contact with. This can be of great importance in terms of the transmission routes of certain diseases and the potential effect on human health, especially in the context of world population growth and environmental pollution. Therefore, the pressing need for strengthening Russia’s position in terms of research and monitoring of airspace (and the microbiological components of bioaerosols in particular) cannot be overestimated.

## References

[1] Jaenicke R. (2005). Science.

[2] Moelling K., Broecker F. (2020). J. Environ. Public. Health. 2020..

[3] Jia Y., Chen Y., Yan P., Huang Q. (2021). Aerosol Air Qual. Res..

[4] Naqvi H.R., Datta M., Mutreja G., Siddiqui M.A., Naqvi D.F., Naqvi A.R. (2021). Environ. Pollut..

[5] Després V.R., Huffman A.J., Burrows S.M., Hoose C., Safatov A.S., Buryak G., Fröhlich-Nowoisky J., Elbert W., Andreae M.O., Pöschl U. (2012). Tellus Ser. B Chem. Phys. Meteorol..

[6] Douwes J., Thorne P., Pearce N., Heederik D. (2003). Ann. Occup. Hyg..

[7] Peccia J., Hernandez M. (2006). Atmos. Environ..

[8] Šantl-Temkiv T., Sikoparija B., Maki T., Carotenuto F., Amato P., Yao M., Morris C.E., Schnell R., Jaenicke R., Pöhlker C. (2020). Aerosol Sci. Technol..

[9] Matthias-Maser S., Jaenicke R. (2000). Atmos. Environ..

[10] Morris C.E., Sands D.C., Bardin M., Jaenicke R., Vogel B., Leyronas C., Ariya P.A., Psenner R. (2008). Biogeosci. Discuss..

[11] Brodie E.L., DeSantis T.Z., Parker J.P.M., Zubietta I.X., Piceno Y.M., Andersem G.L. (2007). Proc. Natl. Acad. Sci. USA..

[12] Xie W., Li Y., Bai W., Hou J., Ma T., Zeng X., Zhang L., An T. (2021). Front. Environ. Sci. Eng..

[13] Puspitasari F., Maki T., Shi G., Chen B., Kobayashi F., Hasegawa H., Iwasaka Y. (2015). Air Quality, Atmosphere & Health..

[14] Safatov A., Agafonov A., Arshinov M., Baklanov A., Belan B., Buryak G., Fofonov A., Generalov M., Kozlov A., Lapteva N. (2018). Atmospheric and Oceanic Optics..

[15] Gong J., Qi J., E B., Yin Y., Gao D. (2020). Environ. Pollut..

[16] Li M., Qi J., Zhang H., Huang S., Li L., Gao D. (2011). Sci. Total Environ..

[17] Park J., Tomoaki I., Masao N., Yamaguchi N. (2016). Sci. Repts..

[18] Janyasuthiwong S., Rungratanaubon T., Saiohai T. (2021). Int. J. Sci. Innov. Technol..

[19] Brągoszewska E., Mainka A., Pastuszka J.S. (2017). Atmosphere..

[20] Shaffer B.T., Lighthart B. (1997). Microb. Ecol..

[21] Dong L., Qi J., Shao C., Zhong X., Gao D., Wan Cao W., Gao J., Bai R., Long G., Chu G. (2016). Sci. Total Environ..

[22] Fahlgren C., Bratbak G., Sandaa R.-A., Thyrhaug R., Zweifel U.L. (2011). Aerobiologia..

[23] Andreeva I.S., Safatov A.S., Puchkova L.I., Emelyanova E.K., Buryak G.A., Ternovoi V.A. (2021). Optika Atmosfery i Okeana..

[24] Safatov A.S., Andreeva I.S., Buryak G.A., Olkin S.E., Reznikova I.K., Belan B.D., Panchenko M.V., Simonenkov D.V. (2022). Atmosphere..

[25] Andreeva I.S., Safatov A.S., Puchkova L.I., Emelyanova E.K., Buryak G.A., Olkin S.E., Reznikova I.K., Ohlopkova O.V. (2019). Bulletin of Nizhnevartovsk State University..

[26] Shammi M., Rahman M.M., Tareq S.M. (2021). Front. Environ. Sci..

[27] Georgakopoulos D.G., Després V., Frohlich-Nowoisky J., Psenner R., Ariya P.A., Pósfai M., Ahern H.E., Moffett B.F., Hill T.C.J. (2009). Biogeosciences..

[28] Peccia J., Milton D.K., Reponen T., Hill J. (2008). Environ. Sci. Technol..

[29] Deguillaume L., Leriche M., Amato P., Ariya P. A., Delort A.-M., Pöschl U., Chaumerliac N., Bauer H., Flossmann A.I., Morris C.E. (2008). Biogeosci. Discuss..

[30] Christner B.C., Morris C.E., Foreman C.M., Cai R., Sands D.C. (2008). Science..

[31] Amato P., Menager M., Sanseime M., Laj P., Mailhot G., Delort A.M. (2005). Atmos. Environ..

[32] Pratt K.A., DeMott P., French J., Wang Z., Westphal D.L., Heymsfield A.J., Twohy C.H., Prenni A.J., Prather K.A. (2009). Nat. Geosci..

[33] Yoo K., Lee T.K., Choi E.J., Yang J., Shukla S.K., Hwang S.I., Park J. (2017). J. Environ. Sci..

[34] Lierl M.B., Hornung R.W. (2003). Ann. Allergy Asthma Immunol..

[35] Dales R.E., Cakmak S., Burnett R.T., Judek S., Coates F., Brook J.R. (2000). Am. J. Respir. Crit. Care Med..

[36] Rosas I., Salinas E., Yela A., Calva E., Eslava C., Cravioto A. (1997). Appl. Environ. Microbiol..

[37] Henningson E.W., Ahlberg M.S. (1994). J. Aerosol Sci..

[38] Su X., Sutarlie L., Loh X.J. (2020). Chem. Asian. J..

[39] Lim J.H., Nam S.H., Kim J., Kim N.H., Park G.S., Maeng J.S., Yook S.J. (2022). J. Biomech. Eng..

[40] Garrido-Cardenas J.A., Manzano-Agugliaro F. (2017). Curr. Genet..

[41] Shin S.K., Kim J., Ha S.M., Oh H.S., Chun J., Sohn J., Yi H. (2015). PLoS One..

[42] Marchesi J.R., Ravel J. (2015). Microbiome..

[43] Hou J., Sievert S.M., Wang Y., Seewald J.S., Natarajan V.P., Wang F., Xiao X. (2020). Microbiome..

[44] Osborne P., Hall L.J., Kronfeld-Schor N., Thybert D., Haerty W. (2020). Environmental Microbiome..

[45] Bashir A.K., Wink L., Duller S., Schwendner P., Cockell C., Rettberg P., Mahnert A., Beblo-Vranesevic K., Bohmeier M., Rabbow E. (2021). Microbiome..

[46] Zhou X., Leite M.F.A., Zhang Z., Tian L., Chang J., Ma L., Li X., van Veen J.A., Tian C., Kuramae E.E. (2021). Environmental. Microbiome..

[47] Bowers R.M., McCubbin I.B., Hallar A.G., Fierer N. (2012). Atmos. Environ..

[48] Bowers R.M., Clements N., Emerson J.B., Wiedinmayer C., Hannigan M.P., Fierer N. (2013). Environ. Sci. Technol..

[49] Bertolini V., Gandolfi I., Ambrosini R., Bestetti G., Innocente E., Rampazzo G., Franzetti A. (2013). Appl. Microbiol. Biotechnol..

[50] DeLeon-Rodriguez N., Lathem T.L., Rodriguez-R L.M., Barazesh J.M., Anderson B.E., Beyersdorf A.J., Ziemba L.D., Bergin M., Nenes A., Konstantinidis K.T. (2013). PNAS..

[51] Serrano-Silva N., Calderon-Ezquerro M.C. (2018). Environ. Pollut..

[52] Mu F., Li Y., Lu R., Qi Y., Xie W., Bai W. (2020). Atmosph. Res..

[53] Cao C., Jiang W., Wang B., Fang J., Lang J., Tian G., Jiang J., Zhu T. (2014). Environ. Sci. Technol..

[54] Xu J. (2016). Genome..

[55] Deiner K., Bik H.M., Machler E., Seymour M., Lacoursiere-Roussel A., Altermatt F., Creer S., Bista I., Lodge D.M., de Vere N. (2017). Mol. Ecol..

[56] Luhung I., Uchida A., Lim S.B.Y., Gaultier N.E., Kee C., Lau K. J. X., Gusareva E.S., Heinle C.E., Wong A., Balakrishnan N. V. (2021). npj Biofilms Microbiomes..

[57] Wang Z., Li J., Qian L., Liu L., Qian J., Lu B., Guo Z. (2019). J. Vis. Exp..

[58] Gusareva E.S., Gaultier N.P.E., Premkrishnan B.N.V., Kee C., Lim S. B.Y., Heinle C. E., Purbojati R.W., Nee A.P., Lohar S.R., Yanqing K. (2020). Sci. Rep..

[59] Fan X.-Y., Gao J.-F., Pan K.-L., Li D.-C., Dai H.-H., Li X. (2019). Environ. Pollut..

[60] Li W., Yang J., Zhang D., Li B., Wang E., Yuan H. (2018). Front. Microbiol..

[61] Ruiz-Gil T., Acuña J. J., Fujiyoshi S., Tanaka D., Noda J., Maruyama F., Jorquera M.A. (2020). Environ. Int..

[62] Tang K., Huang Z., Huang J., Maki T., Zhang Sh., Ma X., Shi J., Jianrong B., Zhou T., Wang G. (2017). Atmospheric Chemistry and Physics Discussions..

[63] Pollegioni P., Mattioni C., Ristorini M., Occhiuto D., Canepari S., Korneykova M.V., Gavrichkova O. (2022). Atmosphere..

[64] Els N., Greilinger M., Reisecker M., Tignat-Perrier R., Baumann-Stanzer K., Kasper-Giebl A., Sattler B., Larose C. (2020). Front. Microbiol..

[65] González-Martín C., Pérez-González C.J., González-Toril E., Expósito F.J., Aguilera Á., Díaz J.P. (2021). Front Microbiol..

[66] Yamaguchi N., Park J., Kodama M., Ichijo T., Baba T., Nasu M. (2014). Microb. Environ..

[67] De Deckker P., Munday C.I., Brocks J., O’Loingsigh T., Allison G.E., Hope J., Norman M., Stuut J., Tapper N., Kaars S.V.D. (2014). Aeolian Res..

[68] Maki T., Kobayashi F., Yamada M., Hasegawa H., Iwasaka Y. (2013). Aerobiologia..

[69] Maki T., Lee K. C., Kawai K., Onishi K., Hong C. S., Kurosaki Y., Shinoda M., Kai K., Iwasaka Y., Archer S.D.J. (2019). J. Geophys.Res.: Atmospheres..

[70] Meola M., Lazzaro A., Zeyer J. (2015). Front. Microbiol..

[71] Jiang X., Wang C., Guo J., Hou J., Guo X., Zhang H., Tan J., Li M., Li X., Zhu H. (2022). Environ. Men. Sci. Technol..

[72] Maki T., Furumoto Sh., Asahi Yu., Lee K., Watanab K., Aoki K., Murakami M., Tajiri T., Hasegawa H., Mashio A., Iwasaka Y. (2018). Atmosph. Chem. Phys..

[73] Hiraoka S., Miyahara M., Fujii K., Machiyama A., Iwasaki W. (2017). Front. Microbiol..

[74] Zhou F., Ni M., Zhen Y., Su Y., W. Y., Zhu T., Shen F. (2021). J. Aerosol. Sci..

[75] Genitsaris S., Kormas K.A., Moustaka-Gouni M. (2011). Front Biosci..

[76] Trout-Haney J.V., Heindel R.C., Virginia R. A. (2020). Environ. Microbiol. Rep..

[77] Bowers R.M., Lauber C.L., Wiedinmyer C., Hamady M., Hallar A.G., Fall R., Knight R., Fierer N. (2009). Appl. Environ. Microbiol..

[78] Al Salameen F., Habibi N., Uddin S., Al Mataqi K., Kumar V., Al Doaij B., Al)Amad S., Al Ali E., Shirshikhar F. (2020). PLoS One..

[79] Hanson B., Zhou Y., Bautista E.J., Urch B., Speck M., Silverman F., Muilenberg M., Phipatanakul W., Weinstock G., Sodergren E., Gold D. R., Sordillo J.E. (2016). Environ. Sci. Process Impacts..

[80] Maki T., Chen B., Kai K., Kawai K., Fujita K., Ohara K., Kobayashi F., Davaanyam E., Noda J., Minamoto Y., Shi G., Hasegawa H., Iwasaka Y. (2019). Atmosph. Environ..

[81] Rodó X., Curcoll R., Robinson M., Ballester J., Burns J.C., Cayan D.R., Lipkin W.I., Williams B.L., Couto-Rodriguez M., Nakamura Y., Uehara R., Tanimoto H., Morguí J.A. (2014). Proc. Natl. Acad. Sci. USA..

[82] Rosa L.H., Pinto O., Convey P., Carvalho-Silva M., Rosa C.A., Câmara P. (2021). Microb. Ecol..

[83] Yang T., Han Y.P., Li L., Liu J.X. (2019). Huan Jing Ke Xue..

[84] Nageen Y., Asemoloye M.D., Põlme S., Wang X., Xu S., Ramteke P.W., Pecoraro L. (2021). BMC Microbiol..

[85] Hanson M., Petch G.M., Ottosen T.-B., Skjøth C.A. (2022). Sci. Total Environ..

[86] Tang K., Sánchez-Parra B., Yordanova P., Wehking J., Backes A. T., Pickersgill D. A., Maier S., Sciare J., Pöschl U., Weber B., Fröhlich-Nowoisky J. (2022). Biogeosciences..

